# Case Report: a 28-year-old female patient presented with recurrent fevers and episodes of shock due to *ZBTB24* pathogenic variant

**DOI:** 10.3389/fimmu.2026.1703785

**Published:** 2026-03-04

**Authors:** Jie Gao, Xiaoling Jiang, Ye Tian, Rui Zhai, Yan Chen, Jinling Guo, Liulin Wu, Mei Hu

**Affiliations:** 1 Department of Critical Care Medicine, The Ninth Medical Center of Chinese People’s Liberation Army General Hospital, Beijing, China; 2 Department of Critical Care Medicine, Meishan Cancer Hospital, Sichuan Meishan, Meishan, China; 3 Key Laboratory of Environmental Sense Organ Stess and Health, Ministry of Ecology and Environment, Beijing, China; 4 Outpatient Department, The Ninth Medical Center of Chinese People’s Liberation Army General Hospital, Beijing, China

**Keywords:** case report, immunodeficiency, immunodeficiency-centromeric instability-facial anomalies syndrome type 2 (ICF2), sepsis, septic shock, *ZBTB24*

## Abstract

**Background:**

Immunodeficiency, Centromeric Instability, and Facial Anomalies Syndrome, commonly known as ICF syndrome, is a rare multisystem autosomal recessive disorder. ICF syndrome is primarily classified into five subtypes: ICF1, ICF2, ICF3, ICF4, and ICFX. Among these, the ICF2 subtype is mainly caused by pathogenic variant in the *ZBTB24*.

**Case presentation:**

A 28-year-old female patient was admitted to our hospital presenting with fever and shock. Despite aggressive antimicrobial therapy, the patient continued to experience repeated episodes of infectious shock following admission for sepsis. This abnormality drew the doctors’ attention and sparked in-depth discussion and analysis. With the discovery of abnormalities in the patient’s immune cells, we became even more convinced that the underlying cause might be a genetic pathogenic variant in the patient. Ultimately, after conducting whole exome sequencing, we identified a homozygous pathogenic variant in the *ZBTB24* (chr6:109476256 G>A, NM_014797.3: c.1123C>T, p.Gln375*) in the patient.

**Result:**

Based on literature review, we implemented a treatment regimen of gamma globulin (10 g/day) combined with antibiotics for the patient. Our efforts ultimately proved successful: the patient recovered fully and was discharged from the hospital. During the one-year follow-up, the patient remained in good condition.

**Conclusion:**

The therapeutic regimen of gamma globulin combined with antibiotics has demonstrated beneficial effects in the treatment of our reported patient.

## Introduction

Immunodeficiency, Centromeric Instability, and Facial Anomalies Syndrome (ICF syndrome) is a rare multisystem autosomal recessive genetic disorder (1). Patients with ICF syndrome typically present with immunodeficiency and hypogammaglobulinemia, leading to recurrent and potentially life-threatening respiratory and gastrointestinal infections, often resulting in hospitalization before the age of two (2, 3). Additionally, they often exhibit distinct facial abnormalities, including a flattened nasal bridge, widely spaced eyes, low-set ears, among other features (3). ICF syndrome is primarily classified into five subtypes: ICF1, ICF2, ICF3, ICF4, and ICFX (4). ICF1, caused by homozygous or compound heterozygous pathogenic variant in the DNA methyltransferase 3B (*DNMT3B*) gene, accounts for approximately 50% of reported ICF cases (5). ICF2, resulting from homozygous or compound heterozygous pathogenic variant in the zinc finger and BTB domain-containing protein 24 (*ZBTB24*), comprises about 30% of documented ICF cases (6). ICF3 is caused by pathogenic variant in the cell division cycle associated 7 (*CDCA7*) gene, while ICF4 is attributed to pathogenic variant in the HELicase lymphoid specific (*HELLS*) gene (4). In recent years, a small number of cases with unknown pathogenic genes have been identified and are referred to as ICFX syndrome (7).

ZBTB24 belongs to the ZBTB protein family and works together with HELLS and CDCA7 to maintain DNA methylation in intergenic regions and repetitive elements (8). In mice, deletion of the BTB domain within *ZBTB24* leads to early embryonic lethality (9). ZBTB24 plays a critical role in regulating B cell differentiation by promoting heme synthesis (10). Individuals with *ZBTB24* pathogenic variant exhibit progressive depletion of B cells and CD4+ T cells, resulting in immunodeficiency (1, 11). Notably, *ZBTB24* pathogenic variant frequently is nonsense pathogenic variant, leading to hypogammaglobulinemia (5). Consequently, affected individuals are prone to recurrent and potentially life-threatening respiratory and gastrointestinal infections (2).

In our case, the patient experienced recurrent episodes of fever and shock, which prompted us to consider conducting immunocyte examinations. The patient’s initial immunophenotyping analysis revealed an abnormally significant reduction in NK and B cell populations, accompanied by a notable elevation in T cell counts, particularly a pronounced expansion of CD8+ T cells. This pattern deviates from the conventional immunological alterations typically associated with sepsis. This finding prompted the clinicians to consider the possibility of an underlying immunodeficiency disorder in the patient. After observing abnormal facial features in the patient, the doctors ultimately identified a homozygous pathogenic variant in the *ZBTB24* through whole-exome sequencing (WES).

## Case presentation

The patient, a 28-year-old female, was admitted to our hospital, presenting with “fever and shock for five days”. The patient had previously undergone surgical interventions for strabismus and trichiasis at the age of three. Thirteen years ago, she was hospitalized due to pleural effusion, and she underwent a cesarean section three years ago. Her immediate family members, including her daughter and parents, are in good health.

Five days prior to hospital admission, the patient experienced a fever with a peak temperature of 39.4 °C, accompanied by coughing and sputum production. The following day, the patient exhibited significant periorbital edema in the right eye, accompanied by rapidly progressive dyspnea, and subsequently fell into a coma. The patient was transported to the emergency department of the local hospital. In light of the patient’s critically low levels of white blood cells, red blood cells, and platelets, as well as the development of coma, respiratory failure, and shock, tracheal intubation and mechanical ventilation were promptly initiated. Empirical treatment with Meropenem and Linezolid, along with hemopurification, was administered in the emergency department. During the 48-hour period that followed, the patient’s condition demonstrated a progressive deterioration. Consequently, on the fifth day post-initial assessment, she was transferred to our facility.

**Figure 1:**
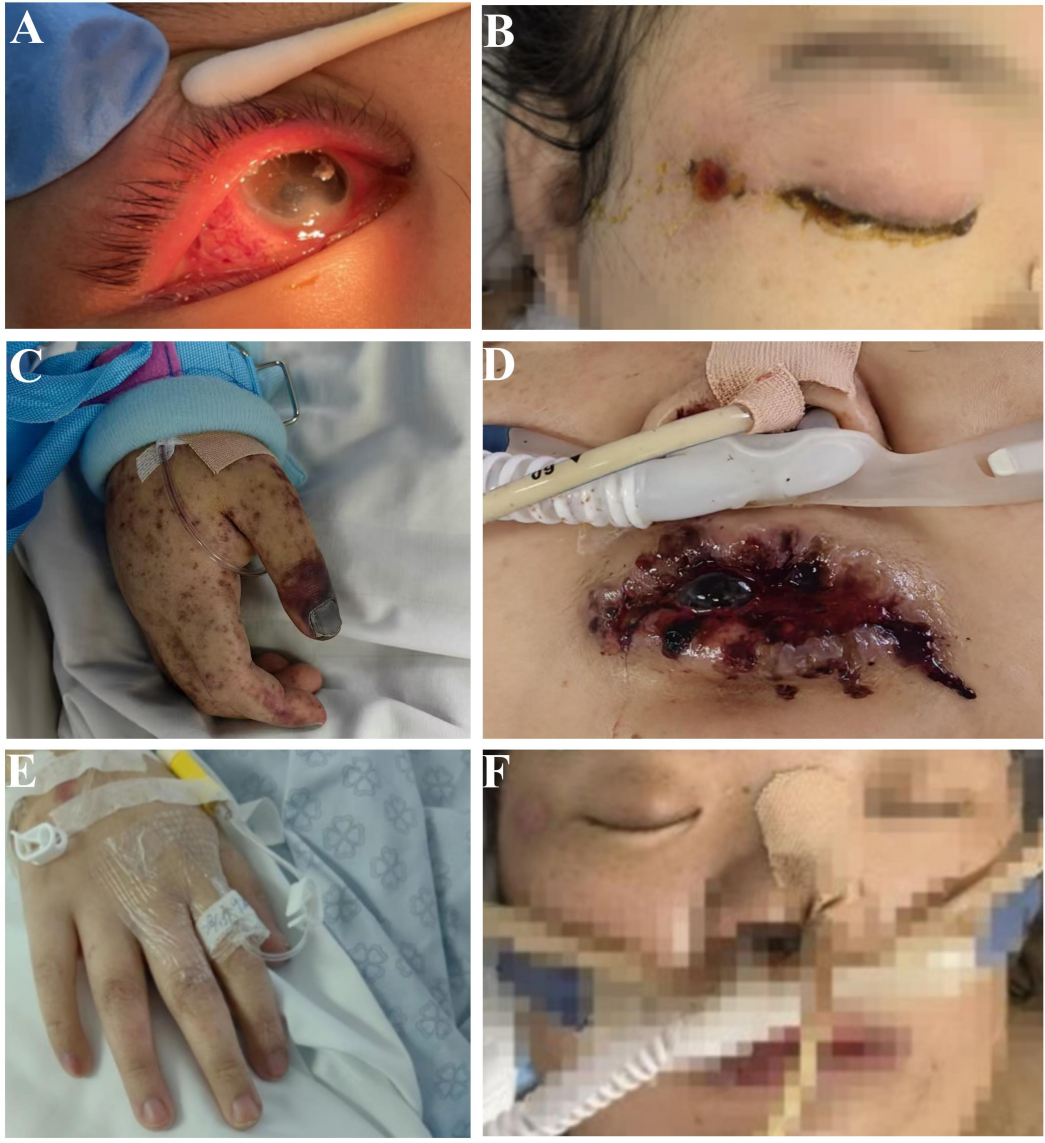
Patient characterisation. Upon admission, the patient presented with purulent discharge, eyelid edema and conjunctival hyperemia in the right eye **(A)**. There was a ulceration near the right eye canthus **(B)**. Cyanosis of the fingers and fingernails **(C)**. On the ninth day of ICU admission, the patient had herpes around the lips **(D)**. On the 42nd day of ICU admission, the patient’s fingers **(E)** and face **(F)** returned to normal.

Upon clinical assessment, the patient was found to be in a comatose state with a body temperature of 38.5 °C and blood pressure of 80/60 mmHg, which was maintained through continuous infusion of Norepinephrine at 0.5 μg/kg/min and Metaraminol at 8 μg/kg/min. Key physical findings included eyelid edema and purulent discharge from the right eye. The patient presented with herpetic lesions on the face, perioral region, lips, and lower extremities, some of which exhibited ulceration (**Figures 1A, B**). Additionally, purpuric macules were observed on the chest, back, and extremities. The patient also demonstrated cyanosis of the fingers and fingernails (**Figure 1C**). Mechanical ventilation was initiated in SIMV mode with the following settings: pressure support at 12 cm H_2_O, respiratory rate of 21 breaths per minute, tidal volume of 380 ml, oxygen concentration of 40%, and oxygen saturation maintained at 90%. On auscultation, the lungs exhibited rough breath sounds. Metagenomic next-generation sequencing (mNGS) identified Haemophilus influenzae in both sputum and blood samples. The SOFA score was 18, and the initial diagnosis was septic shock.

**Figure 2:**
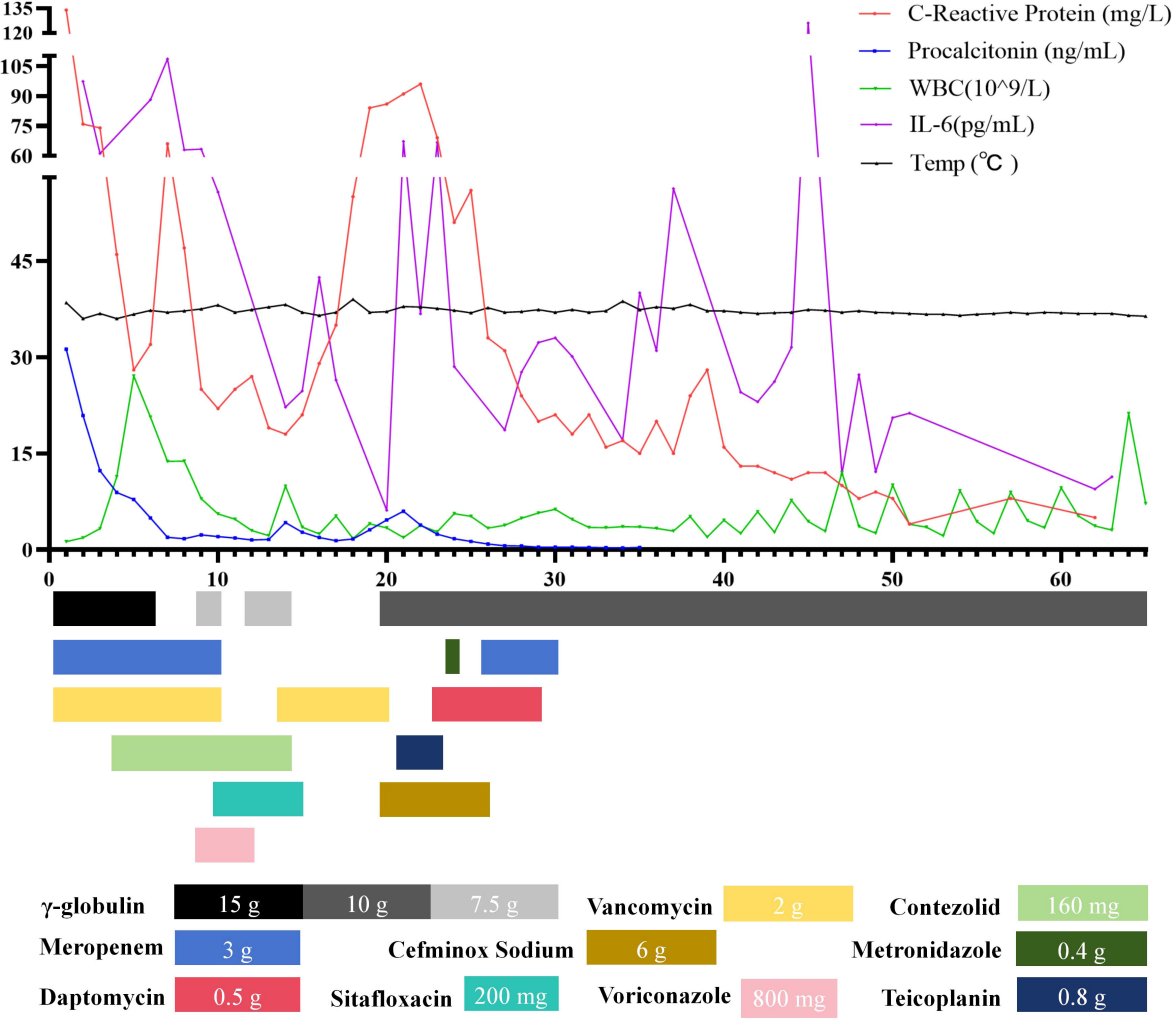
A flowchart of the patient’s laboratory data and treatment plan. The upper section displays the patient’s clinical and laboratory data in the ICU, while the lower section presents the daily treatment regimen and dosage information.

**Figure 3:**
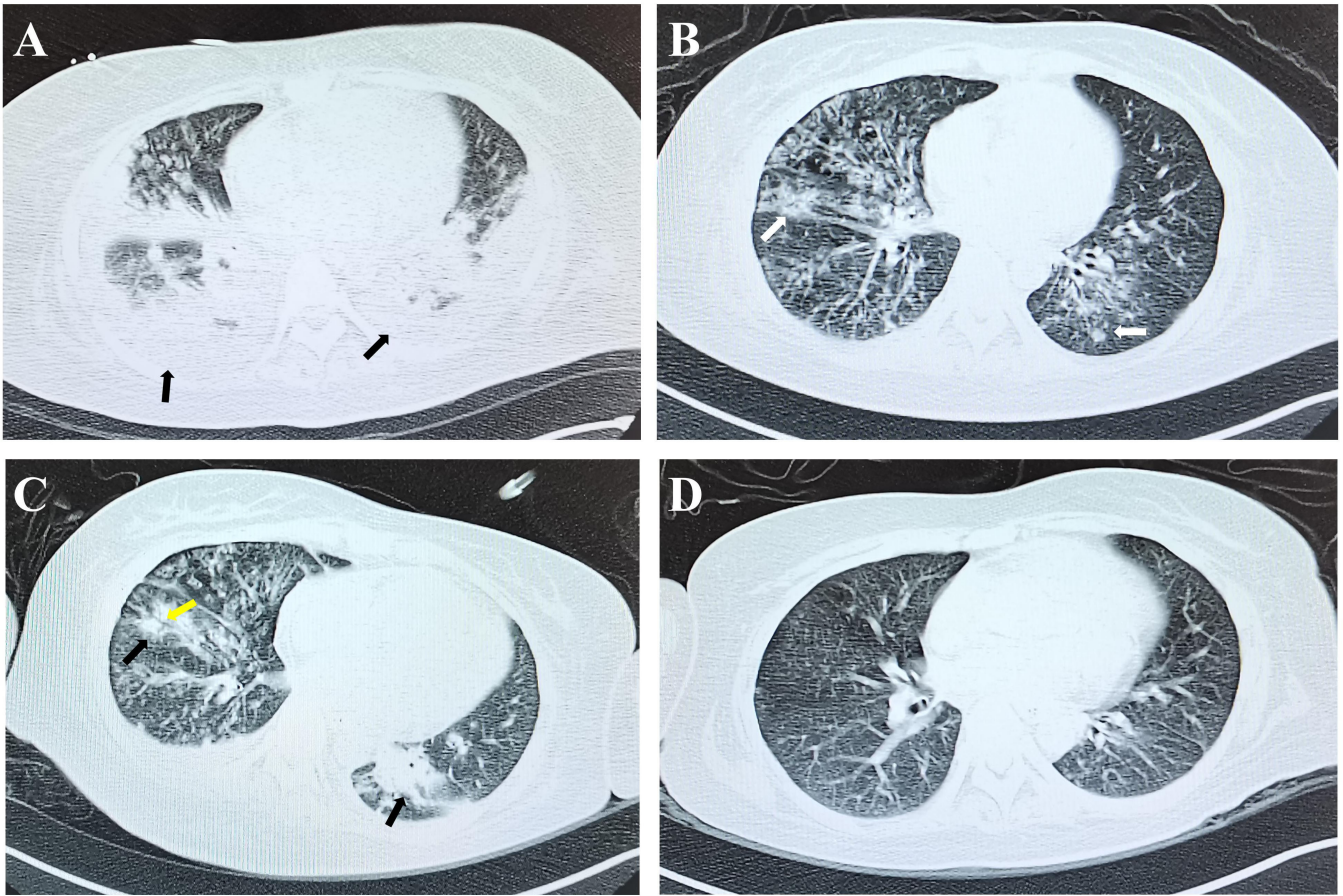
Patient’s chest CT results. Upon admission, the patient’s chest CT revealed lung consolidation (black arrows, **(A)**. On the eighth day of ICU admission, the consolidation resolved, presenting as ground - glass opacities (white arrows, **(B)**. On the 18th day of ICU admission, the patient’s condition had deteriorated again. The consolidation in both lungs had increased (black arrows), and air-space consolidation had emerged (yellow arrow, **(C)**. On the 60th day of ICU admission the patient’s lungs were normal **(D)**.

Abnormalities were observed in liver function, kidney function, and coagulation parameters (**Table 1**). Furthermore, there was a notable elevation in the white blood cell count (WBC), C-reactive protein (CRP), Interleukin-6 (IL-6) and procalcitonin (PCT) levels (**Figure 2**; **Table 1**). Bone marrow aspiration revealed an infection and ruled out leukemia. The patient exhibited elevated troponin levels. Electrocardiogram and bedside ultrasound revealed limb lead low voltage, T-wave abnormalities, multifocal atrial tachycardia, and segmental wall motion abnormalities, all of which are indicative of stress cardiomyopathy. A chest CT scan revealed bilateral patchy areas of consolidation at the level of the lower lobes (**Figure 3A**). mNGS identified Haemophilus influenzae and Staphylococcus aureus in both bronchoalveolar lavage fluid (BALF) and blood samples. Both strains had previously been subjected to antibiotic therapy. However, considering the patient’s low platelet count (**Table 1**) linezolid was replaced with vancomycin. And considering her infection condition, the doctor administered γ-globulin therapy. We developed a timeline of the anti-infection regimen, as shown in **Figure 2**. Concurrently, fluid resuscitation with both colloid and crystalloid solutions was maintained. To manage the resistant bacterial infection in the eye, treatment was initiated with vancomycin ophthalmic solution and dexamethasone ophthalmic solution.

**Table 1:** Laboratory findings. Serial monitoring of blood parameters demonstrated initial abnormalities on admission, worsening during exacerbations (day 8 and day 18), and progressive normalization by discharge.

Variable	Reference range, this hospital†	Admission	Hospital day 5	Hospital day 17	Discharge
White Blood Cell (×10^9^/L)	3.50-9.50	1.87	13.75	55.0	7.18
Red Blood Cell (×10^12^)	3.80-5.10	2.17	2.96	1.74	3.67
Hemoglobin (g/L)	115-150	64	87	99	109.0
Platelet (×10^9^/L)	125.0-350.0	42.0	40.0	34.2	152.0
B(cell/µL)	90-580	-	-	0	1
Absolute Neutrophil Count (×10^9^/L)	1.80-6.30	1.08	13.05	1.56	5.08
Absolute Lymphocyte Count (×10^9^/L)	1.10-3.20	0.69	0.4	0.14	1.3
Eosinophil Ratio (%)	0.4-8.0	0.0	0.1	0.04	4.24
Alanine Aminotransferase (U/L)	7.0-40.0	496.7	94.0	48.0	20.2
Aspartate Aminotransferase (U/L)	13.0-35.0	3475.0	178.0	90.0	55.0
Total Protein (g/L)	65.0-85.0	44.8	71.4	64.9	79.4
Albumin (g/L)	40.0-55.0	54.9	51.5	36.3	2.9
Globulin (g/L)	20.0-40.0	21.3	17.0	13.3	3.5
Total Bilirubin (µmol/L)	0-23.0	2086	371	431	261
Direct Bilirubin (µmol/L)	0-8.0	548	66	33	13
Indirect Bilirubin (µmol/L)	0-15.0	1836	171	1607	149
Lactate Dehydrogenase (U/L)	120-246	24.9	71.6	89.3	55.1
Creatine Kinase (U/L)	30-135	24.3	20.1	12.4	17.2
Brain Natriuretic Peptide (pg/mL)	<100	65.7	50.3	44.1	31.9
Prothrombin Time Activity (%)	70.0-130.0	0.7	1.37	1.49	2.51
Thrombin Time (s)	14.0-21.0	17.10	7.16	5.08	0.6
Actived Partial Thrombolastin Time (s)	23.3-32.5	54.9	51.5	36.3	2.9
Fibrinogen (g/L)	1.8-3.5	21.3	17.0	13.3	3.5
D-Dimer (mg/mL)	<0.55	2086	371	431	261
IgA (g/L)	1.0-4.2	-	-	-	0.87
IgM (g/L)	0.5-2.8	-	-	-	0.19
IgG (g/L)	8.6-14.7	-	-	-	32.6
Complement C3(g/L)	0.7-1.4	0.21	-	-	-
Complement C4(g/L)	0.1-0.4	0.05	-	-	-

†Reference values are influenced by many variables, including the patient population and the laboratory methods used.

The patient demonstrated elevated D-dimer levels. Thromboprophylaxis was administered through the use of Nadroparin Calcium in combination with plasma supplementation. Additionally, the patient exhibited a significant upregulation of inflammatory cytokines (**Figure 2**), which required the continuation of continuous renal replacement therapy (CRRT).

On the fourth day of ICU admission, Methicillin-resistant Staphylococcus aureus (MRSA) was identified.

On the eighth day of ICU admission, the patient regained consciousness. Blood oxygen levels and blood pressure were within the normal range. Urine output had returned to normal, and spontaneous respiratory effort was notably robust. Inflammatory markers, including CRP, PCT, and cytokines, exhibited significant improvement as detailed in **Figure 2**. A follow-up CT scan demonstrated substantial resolution of lung consolidation (**Figure 3B**). The patient was successfully weaned off mechanical ventilation and extubated. After discontinuation of CRRT, urine output exceeded 1000 mL over a 24-hour period.

On the ninth day of ICU admission, the patient developed herpes lesions around the lips (**Figure 1D**). mNGS identified Aspergillus flavus in the blood.

On the 15th day of ICU stay, the patient experienced intermittent convulsions; however, an urgent head CT scan showed no abnormalities.

On the 18th day of ICU admission, the patient developed a persistent fever exceeding 39 °C (**Figure 2**), remained in a coma, and exhibited signs of respiratory failure and shock. Repeat endotracheal intubation and mechanical ventilation were initiated. A follow-up chest CT revealed new patchy consolidations and additional right-sided pleural effusions (**Figure 3C**). Laboratory results indicated low lymphocyte levels and a resurgence in inflammatory markers (**Table 1**; **Figure 2**). mNGS performed on the BALF identified Haemophilus influenzae, Acinetobacter baumannii, Klebsiella pneumoniae, and Aspergillus flavus. Blood cultures confirmed the presence of Klebsiella pneumoniae.

**Figure 4:**
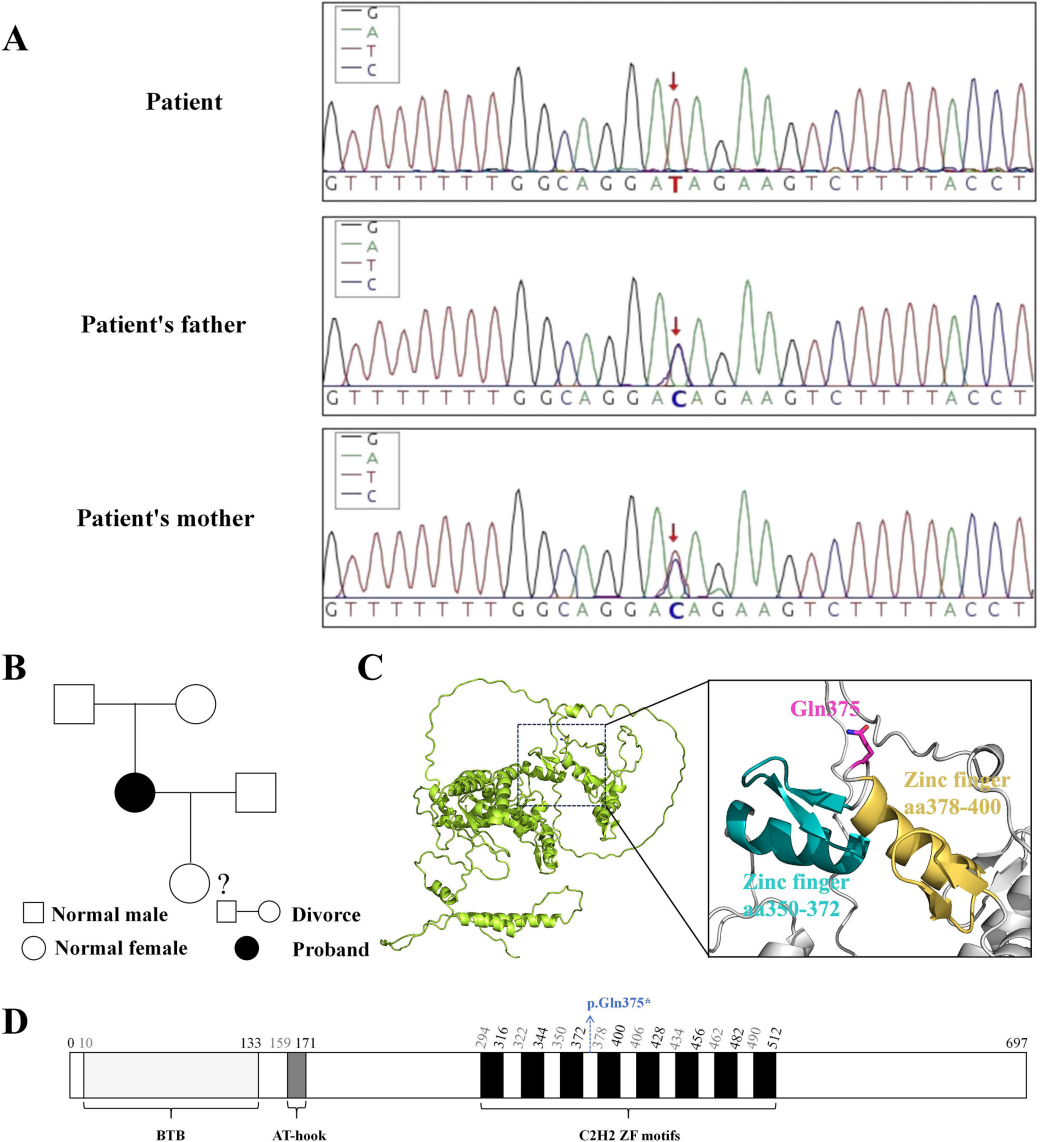
Patient’s *ZBTB24* Gene Information. **(A)** The patient has a homozygous *ZBTB24* gene pathogenic variant (TT, red arrow), with both parents being heterozygous for this variant (CT, red arrow). **(B)** Pedigree of the patient’s family. The genetic information of the patient’s daughter is unknown; **(C)** Structural diagram of ZBTB24, with the enlarged section showing the structural region of the variant site where translation is prematurely terminated. **(D)** Schematic of ZBTB24 Structure. * indicates translation termination.

Despite aggressive antibiotic treatment, the patient continues to experience recurrent fevers, which puzzles the doctor and prompts a thorough search for the underlying cause. Although her parents reported that she had been cognitively capable prior to this illness, the doctor, through careful observation, notices facial abnormalities in the patient (such as round face, a broad and flat nasal bridge, and low-set ears; **Supplementary Figure 2**). Combined with the patient’s condition, the doctor suspects she may have a genetic disorder. The immune cell detection results revealed abnormalities in multiple types of immune cells in the patient, with B lymphocytes accounting for 0.14% and T lymphocytes accounting for 99.39% (**Supplementary Figures 1A–F**). After communicating with the patient’s family and obtaining their consent, a Whole exome sequencing (WES) test was conducted on the patient. WES identified a homozygous pathogenic variant in the zinc-finger- and BTB-domain-containing 24 (*ZBTB24*) ((GRCH38)chr6:109476256 G>A, NM_014797.3: c.1123C>T, p.Gln375*; **Figure 4A**). As shown in **Figures 4C, D**, this pathogenic variant changes the original codon for Gln375 into a stop codon, leading to premature termination of protein translation at Gln375. Pathogenic variant in the *ZBTB24* cause the patient to develop ICF2. Sanger sequencing analysis confirmed that the patient’s parents were heterozygous carriers of the *ZBTB24* pathogenic variant (**Figure 4A**). We constructed a pedigree chart of the patient, as shown in **Figure 4B**. Given the presence of a pathogenic gene pathogenic variant in the patient, a literature review supported the decision to maintain the treatment regimen of gamma globulin combined with antibiotic therapy. The patient was placed under protective isolation, with enhanced disinfection measures and precautions to prevent aspiration. Additionally, we recommended hematopoietic stem cell transplantation for the patient and advised immediate family members to undergo WES for comprehensive genetic evaluation; however, none of these recommendations were adopted.

Over the subsequent two-week period, the patient’s temperature progressively declined), and inflammatory markers exhibited a consistent downward trend (**Figure 2**). Upon achieving hemodynamic stability and successfully passing a spontaneous breathing trial (SBT), the patient was weaned from mechanical ventilation and extubated.

On the 34th day of ICU admission, the patient’s axillary temperature rose to 38.7 °C (**Figure 2**).

On the 42nd day of ICU admission, the patient’s temperature gradually decreased, and her finger and fingernail color returned to normal (**Figure 1**). Additionally, her eyelid edema, purulent discharge, and herpes lesions around her lips resolved (**Figures 1F**). Upon regaining the ability to communicate, it was noted that the patient exhibited signs of cognitive impairment. Her IQ score of 65 indicated a level consistent with intellectual disability.

In the following fortnight, the patient underwent regular infusions of γ-globulin and demonstrated a gradual recovery, marked by stable hemodynamics. Her temperature remained within the normal range (**Figure 2**), and a follow-up chest imaging scan confirmed pulmonary recovery (**Figure 3D**). mNGS analysis of BALF did not identify any microorganisms. Liver function tests, renal function parameters, and coagulation profiles all improved (**Table 1**). Additionally, WBC, CRP, PCT, and IL-6 levels exhibited significant improvement (**Table 1**; **Figure 2**). However, immune cell abnormalities persisted (**Supplementary Figures 1A–F**).

On the 65th day of ICU admission, the patient was discharged and transferred to a rehabilitation hospitals.

## Differential diagnosis

The patient was a 28-year-old married woman who was admitted with a diagnosis of septic shock secondary to severe pneumonia. The bone marrow aspiration revealed an infection. While initial anti-inflammatory treatments showed some efficacy, the patient developed recurrent fevers and episodes of septic shock despite the administration of broad-spectrum antibiotics. These findings raised suspicion of an underlying immunodeficiency disorder, whether primary or secondary in nature. Subsequent hematological evaluations confirmed abnormal immune cell profiles (**Supplementary Figures 1A–F**). Endocrine function tests, glycemic index assessments, and hematologic parameter evaluations all yielded negative results. Furthermore, no iatrogenic factors were detected. Serological tests for AIDS, systemic lupus erythematosus, and rheumatoid arthritiswere also negative. The patient presented with mild facial dysmorphisms, characterized by a round face, a broad and flat nasal bridge, and low-set ears (see **Supplementary Figure 2**), as well as immunodeficiency (**Supplementary Figures 1A–F**). This prompted me to consider the possibility of an underlying genetic condition. Subsequently, her parents acknowledged this concern and agreed to proceed with both genetic testing and an abdominal CT scan.

CT scans of the abdomen, head, and chest revealed no abnormalities. WES identified a pathogenic variant in the patient’s *ZBTB24*, leading to ICF2.

The characteristics of sepsis are excessive hyperinflammation and immune suppression (12). Sepsis-induced immunosuppression involves various cell types, particularly the apoptosis of CD4 T cells, CD8 T cells, B cells, and natural killer (NK) cells, which severely impacts patient health (12, 13). Studies have shown that sepsis patients exhibit a significant reduction in the number of B cells and T cells (14, 15), and this immunosuppressive state may obscure the recognition of pre-existing immune dysfunction in these patients.

## Final diagnosis

ICF2, severe pneumonia, bloodstream infection, sepsis, septic shock, and multi-organ dysfunction.

## Follow up

One year after discharge, the patient showed no respiratory symptoms or fever and was in excellent recovery condition. Post-discharge blood test results indicated normal WBC, RBC, and PLT counts, but the lymphocyte percentage (12.8%) remained low. Her father provided video updates on her recent health status. We recommended regular immunological testing and periodic immunoglobulin supplementation for her. Additionally, the patient may consider undergoing hematopoietic stem cell transplantation therapy. The patient’s family also expressed sincere gratitude to the medical staff.

## Discussion

In accordance with the Sepsis-3 criteria (16), the patient was diagnosed with septic shock and exhibited multiple organ dysfunction syndrome (MODS) affecting the lungs, kidneys, and heart. Despite therapeutic interventions, recurrent fever and episodes of hemodynamic instability persisted, indicating a potential underlying systemic condition. Bone marrow aspiration ruled out the possibility of leukemia. Subsequent examinations revealed abnormalities in the patient’s immune cells, indicating an immunodeficiency. Notably, the doctor identified the patient’s distinctive facial features and cognitive impairment, which had previously been overlooked. Ultimately, a homozygous pathogenic variant in the *ZBTB24* gene was detected in this patient. Nonsense-mediated mRNA decay (NMD) has the potential to degrade RNAs with truncating pathogenic variants that introduce premature termination codons (17). This would lead to loss of ZBTB24 in patients, consequently causing immunodeficiency. The pathogenic variant site identified in this case has not been reported in studies to date.

To date, the literature has reported only a limited number of therapeutic strategies for *ZBTB24* pathogenic variant. A cohort study analysis revealed that the combination of immunoglobulin replacement therapy and antibiotic prophylaxis significantly improved long-term outcomes in patients with ICF syndrome (18). A study reported allogeneic hematopoietic stem cell transplantation for the treatment of ICF syndrome, and suggested that patients receiving transplantation prior to 15 years of age may have more favorable outcomes (19). Currently, research on hematopoietic stem cell therapy for ICF2 syndrome remains relatively limited and requires higher-level evidence for validation.

In conclusion, γ-globulin injections in combination with anti-infective therapies served as the principal treatment modality, resulting in clinically satisfactory outcomes. This case underscores the critical importance of meticulously documenting and investigating all abnormal clinical presentations to identify underlying etiologies. Special attention should be given to patients with recurrent or unusual infections, ensuring that potential genetic factors are thoroughly evaluated. This case also provides valuable insights for clinicians managing patients with *ZBTB24* pathogenic variant associated with infection-related diseases.

## Data Availability

The original contributions presented in the study are included in the article/**Supplementary Material**. Further inquiries can be directed to the corresponding author.

## Supplementary material

Supplementary Figure 1 Results of the patient’s blood immunocytology. The combined depletion of B, NK, and CD4+T cells, coupled with low CD4+/CD8 ratio, strongly suggests a global immunodeficiency state(in each graph, the area between the two purple dashed lines represents the normal reference range).

Supplementary Figure 2 Facial features of the patient. **(A)** Widely-spaced eyes, rounded chin; **(B)** Low-set ears. The ruler in the image is in centimeters.
