# Supplementary figures and images for: Case Report: a 28-year-old female patient presented with recurrent fevers and episodes of shock due to ZBTB24 pathogenic variant
Source: Front Immunol. 2026 Mar 4;17:1703785. doi: 10.3389/fimmu.2026.1703785 (PMC12996111; doi:10.3389/fimmu.2026.1703785)

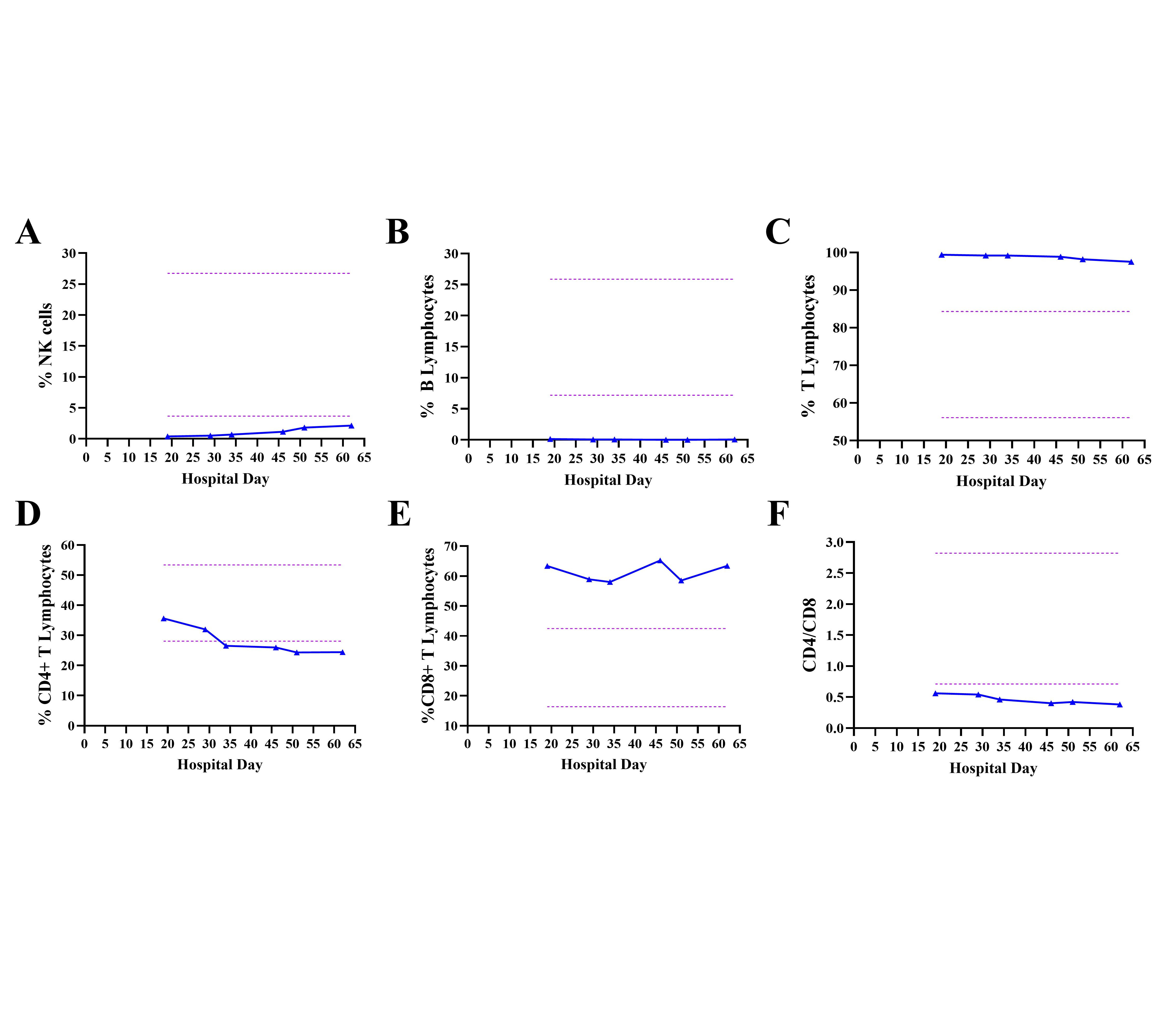

Supplement: Supplementary Figure 1 — Results of the patient’s blood immunocytology. The combined depletion of B, NK, and CD4+T cells, coupled with low CD4+/CD8 ratio, strongly suggests a global immunodeficiency state(in each graph, the area between the two purple dashed lines represents the normal reference range). [file Image1.jpeg]

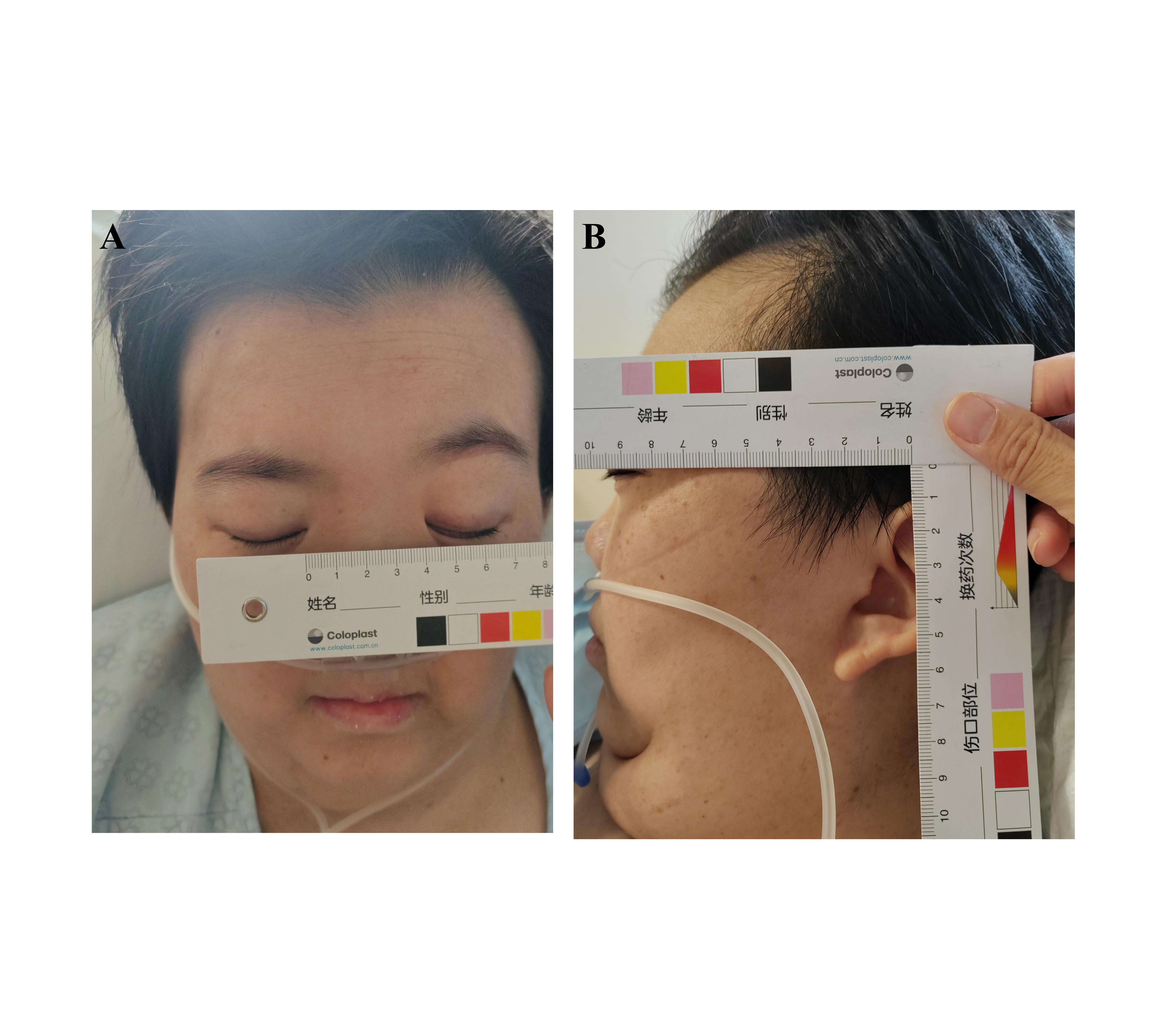

Supplement: Supplementary Figure 2 — Facial features of the patient. (A) Widely-spaced eyes, rounded chin; (B) Low-set ears. The ruler in the image is in centimeters. [file Image2.jpeg]
